# Analysis of the Functional Interaction of Arabidopsis Starch Synthase and Branching Enzyme Isoforms Reveals that the Cooperative Action of SSI and BEs Results in Glucans with Polymodal Chain Length Distribution Similar to Amylopectin

**DOI:** 10.1371/journal.pone.0102364

**Published:** 2014-07-11

**Authors:** Henrike Brust, Tanja Lehmann, Christophe D'Hulst, Joerg Fettke

**Affiliations:** 1 Institute of Biochemistry and Biology, University of Potsdam, Potsdam-Golm, Germany; 2 Unité de Glycobiologie Structurale et Fonctionnelle, Université Lille1, Villeneuve d'Ascq, France; Leibniz-Institute for Vegetable and Ornamental Plants, Germany

## Abstract

Starch synthase (SS) and branching enzyme (BE) establish the two glycosidic linkages existing in starch. Both enzymes exist as several isoforms. Enzymes derived from several species were studied extensively both *in vivo* and *in vitro* over the last years, however, analyses of a functional interaction of SS and BE isoforms are missing so far. Here, we present data from *in vitro* studies including both interaction of leaf derived and heterologously expressed SS and BE isoforms. We found that SSI activity in native PAGE without addition of glucans was dependent on at least one of the two BE isoforms active in Arabidopsis leaves. This interaction is most likely not based on a physical association of the enzymes, as demonstrated by immunodetection and native PAGE mobility analysis of SSI, BE2, and BE3. The glucans formed by the action of SSI/BEs were analysed using leaf protein extracts from wild type and *be* single mutants (*Atbe2* and *Atbe3* mutant lines) and by different combinations of recombinant proteins. Chain length distribution (CLD) patterns of the formed glucans were irrespective of SSI and BE isoforms origin and still independent of assay conditions. Furthermore, we show that all SS isoforms (SSI-SSIV) were able to interact with BEs and form branched glucans. However, only SSI/BEs generated a polymodal distribution of glucans which was similar to CLD pattern detected in amylopectin of Arabidopsis leaf starch. We discuss the impact of the SSI/BEs interplay for the CLD pattern of amylopectin.

## Introduction

Starch is the major storage carbohydrate in plants and forms insoluble granules consisting of two glucose polymers, amylose and amylopectin. In both α-1,4-glycosidic bonds are present but only the latter contains significant amounts of α-1,6-glycosidic bonds - responsible for branching of the glucan chains [1], [2]. Amylopectin, as the major glucose polymer, accounts for 70–80% of the starch and forms a semi-crystalline structure with alternating crystalline and amorphous lamellae having a conserved dimension of 9–10 nm [3], [4]. The basis of this semi-crystalline structure is related to the high order organization of branch points within the starch [5]. Different models exist with regard to the proportion of glucan clustering in amylopectin molecules [6]. Glucan chains have been classified according to the existence and number of branching points thereby A chains represent the outermost chains, whereas B chains connect at least one A or additional B chains. The C chain connects the B chains and is the only chain with a free reducing end [7]. In the currently accepted cluster model of Hizukuri [8] the B chains are sub-grouped with regard to their chain length populations (B1, B2, B3). However, short B1 chains and A chains are responsible for the double helix formation and thereby for the crystalline lamellae. In addition, long chains (B2, B3), which interconnect different clusters, are thought to alternate tangentially or radially [8]–[10].

SSs and BEs are the two enzymes responsible for glycosidic linkage formation during the starch biosynthetic process. In higher plants several isoforms have been reported for both enzyme classes [11]–[13]. Genetic and biochemical studies have revealed distinct functions in starch granule synthesis for the five SS isoforms (AtGBSSI, AtSSI-IV) present in Arabidopsis [14]–[19]. Several SS mutants exhibit alterations in starch content, starch morphology and in most cases specifically different amylopectin chain length distributions (CLD). Transitory starch from Arabidopsis mutants lacking the SSI isoform displayed a reduced amount of short glucan chains (DP 8–12) and was enriched in glucans with chain length of DP 17–20 [14]. Similar results were reported for rice and wheat endosperm [20], [21]. By contrast, SSII deficiency led to an increase of glucan chains with DP 5–11 and a slight decrease of chains longer than DP11 in Arabidopsis [17], [19]. These observations were confirmed for various cereals (wheat [22], barley [23], maize [24], rice [25]). In contrast to the consequences of a deficiency of expression of SSI and SSII, lack of the SSIII isoform revealed only minor effects on the amylopectin CLD [18], [19]. Arabidopsis double mutants lacking either SSI or SSII together with SSIII displayed a more pronounced CLD phenotype, which was characteristic for the respective single mutants and suggests partially overlapping enzymatic functions of the isoforms [17], [19]. However, Arabidopsis plants lacking SSIV displayed only one larger starch granule per chloroplast, indicating participation of SSIV in starch granule initiation. Interestingly, these enlarged granules displayed an identical CLD profile of amylopectin and a similar amylose content compared to wild type [15]. In summary, results from both *in vivo* and *in vitro* studies, using various recombinant SS (rSS) isoforms, support the idea that SSI elongates short outer glucan chains with a DP8–12, whereas SSII extends glucan chains of DP3–8 to establish longer chains with a DP12–35 (DP12–25 in Arabidopsis) and SSIII elongates chains longer than DP25 [16], [17], [25]–[28].

BE isoforms are grouped either to class A (or BEII-type) or B (BEI-type) according to sequence homology and enzymatic properties [29], [30]. In Arabidopsis both BE isoforms (BE2 and BE3, each class A) are functional in amylopectin synthesis, as single knock-outs revealed only weak effects on amylopectin CLD. Thus, it was assumed that both enzymes can substitute for one another. However, for amylopectin synthesis it became apparent that at least one isoform is required since double mutants were unable to synthesis starch [31]. For maize and rice it has previously been reported that BEIIa and BEIIb have distinct functions during starch synthesis in leaf and endosperm, respectively [32]–[36].

Despite the progress in understanding the properties of various starch synthases and branching enzymes, more insight into the cooperative action of both types of enzymes is necessary. It was previously demonstrated that wheat and maize amyloplasts which harbour different starch synthase and branching enzyme isoforms form heteromeric protein complexes. SSI, SSIIa and SSIII isoforms were observed in different combinations with BEIIa and/or BEIIb and the observed protein-protein interactions were stabilized via protein phosphorylation [37]–[40]. One of the best studied multi-subunit complexes consists of three enzymes SSI, BEIIb and SSIIa [41]. Interestingly, when the BEIIb protein was lacking or inactive it was replaced by BEI and/or BEIIa and in some cases in combination with the plastidial glucan phosphorylase [40], [42]. Furthermore, inactive BEIIb prevented the association of the plastidial glucan phosphorylase to the complexes, but not the association of BEI/BEIIa to SSIIa/SSI [42]. In addition, the amylopectin phenotype (CLD) of the corresponding mutant (lacking BEIIb, *amylose extender* mutant) displayed a decreased the number of shorter (<DP15) and increased the number of longer glucan chains with a concomitant reduction of branching. Given that BEI transfers longer glucan chains, the observed phenotype is best explained with regard to the abundance of BEI within the altered protein complex [40], [42].

The above data notwithstanding, information concerning the synergistic action of branching enzymes and starch synthases during glucan formation are lacking. Thus, it is unknown which of the various SS isoforms elongate glucan chains that are branched by the BEs and which of the SS isoforms are able to use these newly formed glucan chains (shortened original chain and transferred chain) as substrate for continued extension. Furthermore, alterations in protein-protein interactions as well as changes in phosphorylation dependencies and/or substitution of specific isoforms can result in structural changes of the starch and/or thereby starch morphology, which are consequently not directly linked to the lacking function of an enzyme. Thus, it is difficult to presume specific functions of the starch synthesizing enzymes.

In summary, we here report on the functional interactions between starch synthase and branching enzyme isoforms from *Arabidopsis thaliana*. We demonstrated that SSI activity in native PAGE is dependent on at least one of the BE-isoforms and independent of external applied glucans. Nevertheless, the reaction is not unprimed in a strict sense as traces of glucans were detected. Furthermore, our results reveal, that all starch synthase isoforms together with BEs were able to form branched glucans, but for each starch synthase isoform interacting with BEs a specific CLD profile was observed. The comparison of these *in vitro* CLD patterns with CLD profile of isolated starch from Arabidopsis wild type leaves showed that only the interaction of SSI and BEs generate the characteristic polymodal CLD profile which is detectable for leaf starch.

## Materials and Methods

### Plant material

*Arabidopsis thaliana* plants were grown under 12 h light (20°C)/12 h dark (16°C) cycle, with light intensity of 150 µmol m^−2^ s^−1^. Relative humidity of 60% was kept constant. SALK lines *AtssII* (At3g01180, SALK_065639), *AtssIII* (At1g11720, SALK_151477) and *AtssIV* (At4g18240, SALK_096130) were purchased from Nottingham Arabidopsis Stock Centre and established in Col-0. *AtssI* (*ss1*-1, At5g24300) and *be* mutants (*Atbe2* [At5g03650, *be2-1*], *Atbe3* [At2g36390, *be3-2*] and *Atbe3/2 [be2-1/be3-2*], respectively) were previously described in Delvallé *et al*. [14] and Dumez *et al*. [31].

If not indicated differently, plant material from five week old plants was harvested in the middle of the light period and immediately frozen in liquid nitrogen.

### Cloning and expression of starch synthase and branching enzyme isoforms

Cloning and expression strategy for rSSI, rSSII, rSSIII, rSSIV, rBE2 and rBE3 was the same as described for recombinant AtSSIII (here rSSIII) in Fettke *et al*. [43]. Primers, restriction sites, and predicted transit peptides are described in Table S1 in File S1. All proteins were expressed without transit peptide using pET23b vector (Novagen) and the *E. coli* strain BL21(DE3). rSSI, rBE2, and rBE3 were also expressed in a glycogen deficient *E. coli* strain *ΔglgCAP* with BL21(DE3) background [16]. For expression, cells were grown in 800 mL (37°C) Luria-Bertani medium containing 100 µg ml^−1^ ampicillin. Culture medium for expression in glycogen deficient *E. coli* strain contained additionally 50 µg ml^−1^ kanamycin and 10 mM glucose. Expression (3 h at 30°C) was induced by addition of isopropylthio-β-galactoside (1 mM final concentration) at OD 600 nm values between 0.4 and 0.6. Harvesting of the cells and purification of His-tagged proteins were performed as previously described [43], except that after loading the column was washed with 25 instead of 15 column volumes. Purified protein fractions were concentrated via ultrafiltration (30 kDa; Amicon Ultra; Millipore) in 50 mM HEPES/NaOH, pH 7.5, 1 mM EDTA, 2 mM DTE and 10% (w/v) glycerol. Aliquots were frozen in liquid nitrogen and stored at −80°C until use. Protein concentration was estimated using Bradford assay kit (SIGMA, Taufkirchen, Germany) with BSA as standard.

### Extraction of soluble buffered proteins

Leaf material was homogenised in liquid nitrogen using mortar and pestle and transferred to centrifuge tubes. Ice-cold buffer (100 mM HEPES/NaOH pH 7.5, 4 mM EDTA, 5 mM DTE, 0.5 mM phenylmethylsulphonyl, 10% [w/v] glycerol) in a ratio of 1∶2.5 ccording to fresh weight of leaf material was added. The homogenate was centrifuged (12 min at 14,000 g, 4°C) and the supernatant was passed through a nylon net (100 µm pore size). Aliquots of the supernatant were frozen in liquid nitrogen and stored at −80°C until use.

### Native PAGE and staining for soluble starch synthase activities

Discontinuous native PAGE was performed as described elsewhere [44]. Two different incubation solutions (a and b) were used to visualize starch synthase activities. The incubation buffer (a) contained 50 mM Tricine/KOH (pH 8.0), 25 mM K-acetate, 5 mM DTE, 2 mM EDTA, 0.025% (w/v) bovine serum albumin, and 1 mM ADPglucose (either purchased from Sigma, Taufkirchen, Germany or synthesised using recombinant AGPase from *E. coli* as described in Fettke *et al*. [44]). Buffer (a) was used for gels that contained 0.02% (w/v) glycogen from oyster (type II, Sigma). Gels without any glucan were incubated with buffer (b) that contained in addition 500 mM Na-citrate (pH 8.0). After electrophoresis the gels were equilibrated for 20 min in the respective buffer, but ADPglucose was omitted. Gels were incubated overnight at room temperature and stained with iodine.

### Native PAGE and staining for starch branching enzyme activities

Analyses of branching enzyme activities were performed using glucan free native gels and the phosphorylase a stimulating assay according to Dumez *et al*. [31].

### 2-Dimensional-PAGE and immunodetection

For re-electrophoresis, native PAGE with different gel concentrations (6.5, 8.0 nd 9.5%) were performed. 80 µg buffer soluble proteins were applied per lane. After electrophoresis gels were cut and stripes were washed two times with water, 15 min each. Gel pieces were denatured in SDS sample buffer (125 mM Tris/HCl, pH 6.8; 4% w/v SDS; 40 mM DTE; 20% w/v glycerol, 0.01% w/v bromphenol blue) at 80°C for 20 min and afterwards placed on the top of the SDS stacking gel and SDS PAGE was performed using separation gel of 7.0% [T] acrylamide/bisacrylamide. Following blotting, nitrocellulose was cut into three pieces according to molecular mass marker and the respective pieces were incubated with a monoclonal mouse antibody raised against AtBE2 and polyclonal rabbit antibodies raised against AtSSI and AtBE3, respectively.

### Extraction of carbohydrates from recombinant protein preparations

For quantification of glucose 150 µg recombinant protein was denatured for 5 min at 95°C and endogenous carbohydrates were hydrolyzed by addition of 0.4 nits amyloglucosidase overnight at 55°C in a total volume of 150 µl (3 mM Na-citrate/citric acid pH 4.9). Following denaturation (5 min at 95°C) and centrifugation (5 min at 21,000 g) glucose amount was estimated using Starch Determination Kit (RBiopharm, Darmstadt, Germany) according to Stitt *et al.* [45].

For chain length distribution analyses 150 µg recombinant protein was filtrated and washed (10 kDa; Amicon Ultra) and the retentate was incubated with 300 nits isoamylase from *Pseudomonas spec*. (Sigma) overnight at 37°C (total volume of 200 µl, 3 mM ammonium-acetate pH 5.5). Following denaturation (5 min at 95°C) samples were filtered (10 kDa) and the filtrates were lyophilised and solubilized in water. HPAEC-PAD analyses was performed as described in Fettke *et al*. [44] except that the solvent was kept in 5 mM acetate (in 100 mM NaOH) for additional 10 inutes prior to elution with linear gradient.

### *In vitro* assays of SS with BE isoforms

Combinations of rSS and rBE isoforms were incubated with equimolar amounts (0.07 nmol for rSSI-IV with rBE2 each and 0.035 nmol for rSSI-IV with rBE3 each) in a total volume of 50 µl (100 mM Na-citrate/citric acid (pH 8.0), 25 mM Tris/HCl (pH 8.0) and 30 mM ADPglucose). Aliquots at different time points were taken and denatured for 4 min at 95°C. For estimation of glucosyl incorporation aliquots were diluted at least two times by addition of 7 mM Na-citrate/citric acid (pH 4.9) and incubated with 0.5 units amyloglucosidase over night at 55°C. Glucose was estimated using Starch Determination Kit. For isoamylase digestion aliquots of the reaction were filtered (3 kDa; Amicon Ultra; Millipore) and the retentates were incubated with 500 units isoamylase at 37°C overnight in total volume of 250 µl. The reaction was stopped by heating (4 min at 95°C) and the digests were again filtrated (10 kDa). The filtrates were lyophilised and resolved in water. Glucose amount was estimated as described before and reducing ends were quantified according to Waffenschmidt and Jaenicke [46].

### Carbohydrate extraction from native PAGE

For analyses of polyglucans formed during incubation with solution (b; see above) buffer soluble proteins of Arabidopsis wild type plants (Ws, Col-0) and mutants *Atbe2* and *Atbe3* were loaded on native PAGE (300 µg per cm lane each). In addition, rSSI in combination with either rBE2 or rBE3 were used (0.2 µg each per cm lane). After incubation (overnight at RT) gels were washed two times with water (15 min each). Gel pieces were homogenized with a pistol. 120 mg gel was incubated with 500 µl of 0.1 M NaOH for 15 min at 95°C, pH was adjusted to pH 5 with 1.75 M acetic acid. 2,500 units isoamylase in 500 µl of 80 mM ammonium-acetate (pH 5.5) were added. Samples were incubated overnight at 37°C under continuous agitation. Following heating (15 min at 95°C) 500 µl water was added and again incubated for 15 min at 37°C with 1,400xrpm in a thermomixer. Following centrifugation (3 min, 20,000×g at RT) the supernatant was collected and gel pieces were again incubated for 15 min (37°C; 1,400xrpm) in 800 µl water. This step was repeated for three times to make sure that the carbohydrates were completely removed from the gel. Supernatants were pooled and deionised using 180 mg of ion exchange resin (Bio-Rad AG 501-X8[D]) prepared in 1 ml tip. The flow through was lyophilized and resolved in water and analysed by HPAEC-PAD according to Fettke *et al*. [44], except that the linear gradient of sodium acetate was extended to 100 min.

### Chain length distribution analysis of carbohydrates produced by interaction of the different SS isoforms with the BEs

rSS isoforms were incubated with either rBE2 or rBE3 and samples were taken from three different time points to get an incorporation of at least 300 to 900 nmol of glucose units per 50 µl reaction volume. Reaction was stopped via heating at 95°C for 4 min. An aliquot was taken for glucose estimation. After filtration (3 kDa) the retentate was incubated with 300 units isoamylase overnight at 37°C. Following denaturation (3 min at 95°C) samples were filtrated (10 kDa), lyophilized, and resolved in water. Glucans (10 nmol reducing ends) were labelled and analysed using CE-LIF as described in Malinova *et al*. [47].

### Extraction and chain length distribution of starch

Starch was extracted from Arabidopsis leaves harvested at the end of light period. Starch was isolated as previously described [48]. Prior to isoamylase digestion starch suspension (1 µg/µl) was solubilised by heating for 5 min at 95°C. 300 µg starch was incubated with 500 units isoamylase (6 mM ammonium-acetate, pH 5.5) in a total volume of 600 µl overnight in a thermomixer at 37°C and 1,400×rpm. Reaction was stopped by heating (95°C for 3 min). Following lyophilisation and resolving in water, 30–45 µg glucose equivalents were applied to HPAEC-PAD.

## Results

### Characterization of starch synthase activities

**Figure 1:**
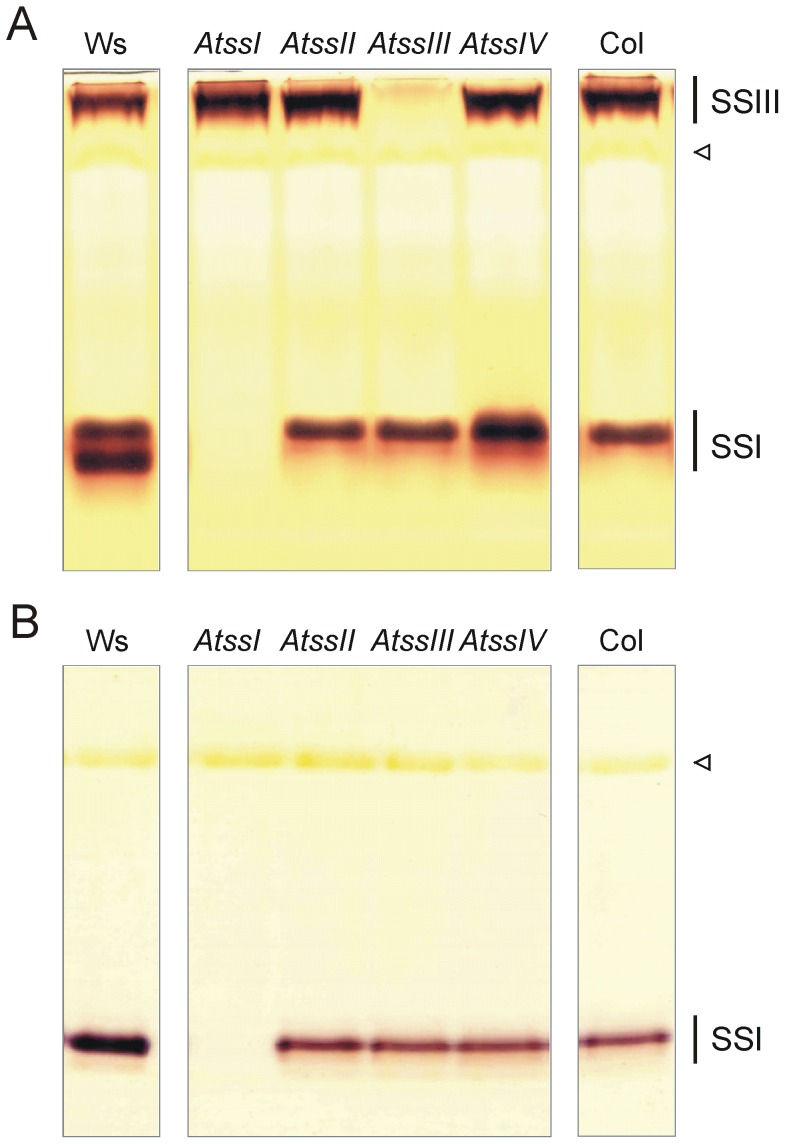
SS activities from different *ss* single knock-out plants. Protein extracts from wild type plants (Ws, Col) and *ss* single knock-out lines (*AtssI* in Ws background, *AtssII, AtssIII* and *AtssIV* in Col-0 background) were electrophoretically separated under non-denaturing conditions (55 µg protein per lane). (A) Separation gel containing oyster glycogen was incubated with 1 mM ADPglucose. (B) Glucan free gel was incubated with 1 mM ADPglucose and in presence of citrate. After Incubation overnight at room temperature gel was washed with water and stained with iodine solution. Positions of SSs are indicated. Rubisco protein appears as yellowish band (open triangle).

**Figure 2:**
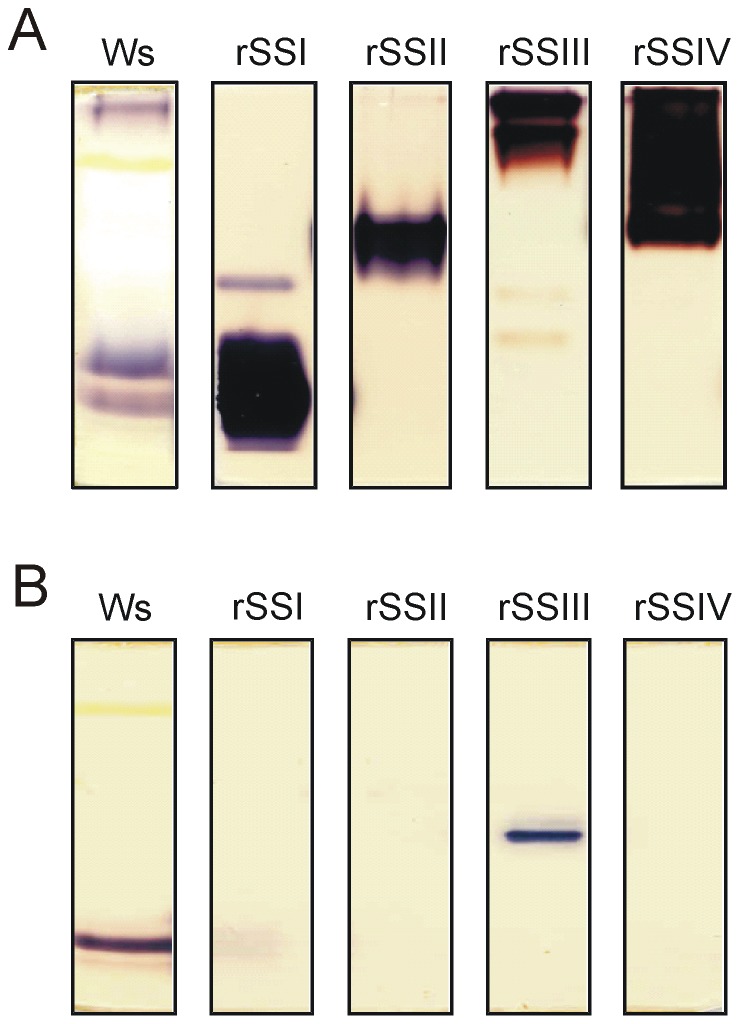
SS activities from recombinant SS isoforms. 0.8 µg of rSSs (rSSI, rSSII, rSSIII and rSSIV) were electrophoretically separated under non-denaturing conditions. (A) Separation gel containing oyster glycogen was incubated with 1 mM ADPglucose. (B) Glucan free gel was incubated with 1 mM ADPglucose in presence of citrate. After incubation overnight at room temperature gels were washed with water and stained with iodine solution.

Starch synthase activity was analysed in Arabidopsis leaf extracts (Fig. 1). In agreement with previous reports [14] only starch synthase isoforms SSI and SSIII activities were detected, even when protein extracts were concentrated and/or fractionated by polyethylene glycol and affinity chromatography (e.g. Blue Sepharose; shows strong binding of SSI and SSIII protein, data not shown). Interestingly, only SSI activity was detected without addition of any glucosyl acceptor like glycogen or soluble starch (Fig. 1B). This activity did not change throughout the diurnal cycle (see Fig. S1 in File S1). Similar analyses were performed with the recombinant isoforms. All SS activities were detected if proteins were heterologously expressed (Fig. 2A). Thereby, rSSI and rSSIII showed a similar mobility in the gel as the corresponding band from leaf extracts (see Fig. 2A).

In contrast to plant extracts, the rSSI protein (and also rSSII and rSSIV) revealed no activities if addition of glucans to the gel was omitted, whereas the rSSIII protein did (Fig. 2B). The latter finding was demonstrated previously [16].

Protein amount of rSSs applied to the gel were probably higher than SSs content in leaf protein extracts. Thus, it is ambiguous, what is the reason for the contradicting observation for SSI activity from plant extract and the heterologous expressed form. Possibly, SSI in contrast to rSSI, contained a glucan serving as a glucosyl acceptor. However, when rSSI was incubated with different glucans (maltodextrin, soluble starch) either with or without ADPglucose prior to electrophoresis no distinct activity band was observed (data not shown). Moreover, no activity was detected (without addition of a glucan acceptor) when using ^14^C-labeled ADPglucose as a more sensitive tool for SS activity detection, even using different buffer systems (see Table S2 in File S1).

### Extracts of be double knock-out plants revealed BE dependent SSI activity

**Figure 3:**
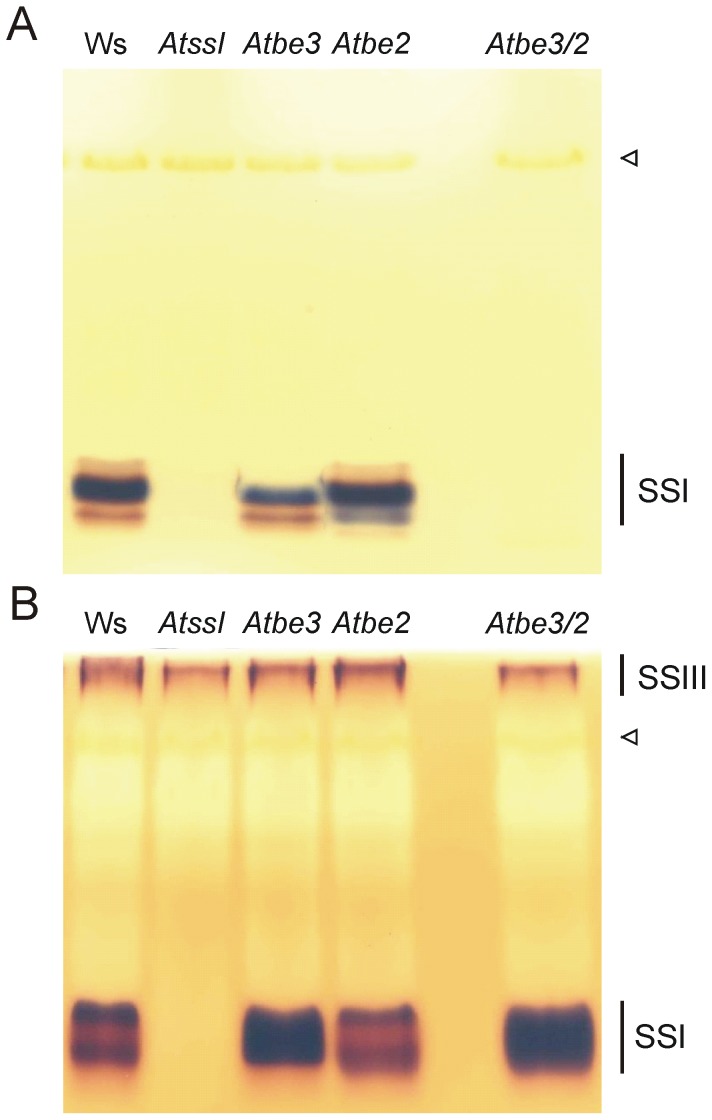
SS activities from different *be* knock-out plants. Leaf protein extracts from *Atbe3*, *Atbe2*, *Atbe3/2* and additionally from wild type (Ws) and *AtssI* were electrophoretically separated under non-denaturing conditions (65 µg protein per lane). (A) Glucan free gel was incubated with 1 mM ADPglucose in presence of citrate (buffer b). (B) Separation gel containing oyster glycogen was incubated with 1 mM ADPglucose (buffer a). After Incubation overnight at room temperature gels were washed with water and stained with iodine solution. Open triangle indicates the position of rubisco.

**Figure 4:**
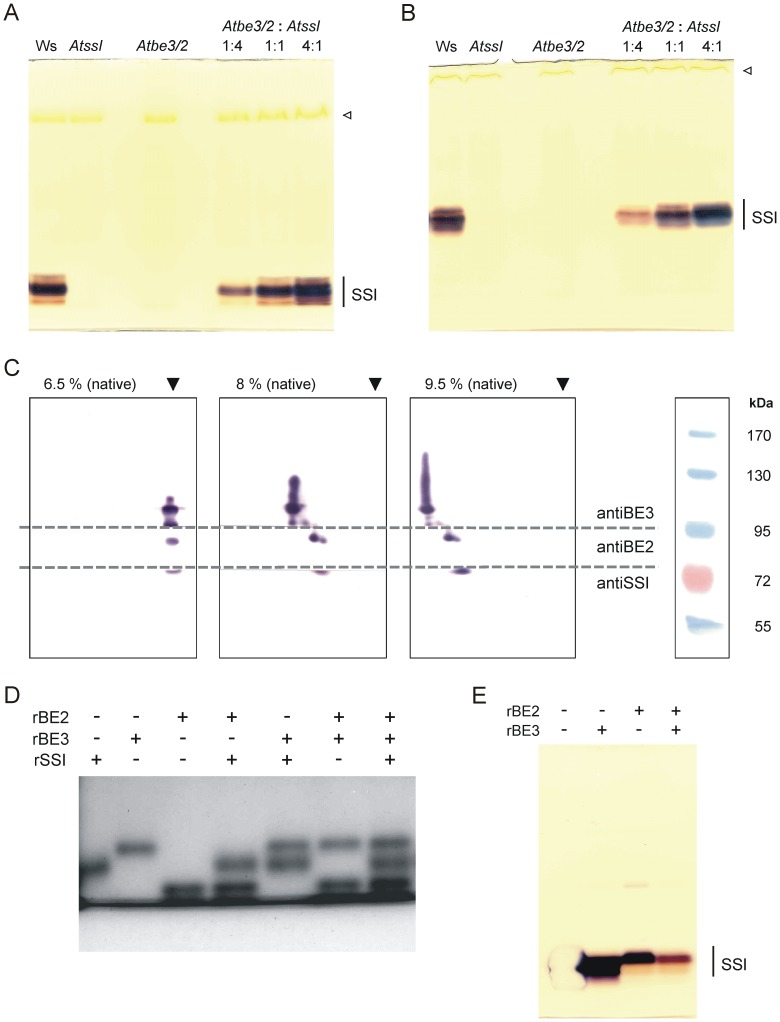
BE dependent SSI activity and mobility of SSI, BE2 and BE3 protein in native PAGE. (A–B) Protein extracts from *be* double knock-out (*Atbe3/2*) and *ss* single knock-out (*AtssI*) lines were mixed prior to electrophoresis using different proportions of protein amounts as indicated (75 µg protein per lane in total). Additionally, protein extracts of the mutants were separately analysed and extracts of wild type (Ws) served as control (65 µg protein per lane). Native PAGE was performed using different separation gel concentrations with either (A) 7.5% [T] or (B) 9.5% [T] acrylamide-bisacrylamide. No glucans were applied to the gel. For SS activity staining, gels were incubated with 1 mM ADPglucose in presence of citrate (buffer b) overnight at room temperature and finally stained with iodine. Open triangle indicates the position of rubisco. (C) Immunodetection of AtSSI, AtBE2 and AtBE3 proteins after re-electrophoresis. Following native PAGE (first dimension) using separation gels with different acrylamide-bisacrylamide concentrations (6.0, 8.0 and 9.5% [T] as indicated) gel stripes were cut and denatured for SDS-PAGE (second dimension). After SDS-PAGE proteins were blotted on nitrocellulose and membranes were cut in three pieces according to molecular weights of AtSSI, AtBE2 and AtBE3. Each of the nitrocellulose pieces were incubated with the respective antibodies (e.g. the nitrocellulose piece with protein marker greater than 95 kDa were incubated with antibody against BE3 whereas the nitrocellulose pieces with protein marker below 72 kDa were incubated with antiSSI). Prestained molecular mass marker served as standard. Black triangles indicate the dye front of the gel stripes from the first dimension. (D) rSSI (0.3 µg) was either mixed in different combinations of rBEs (0.2 µg each) as indicated. Native PAGE and activity staining was performed as described for (A). (E) Proteins (rSSI, rBE2 and rBE3) were mixed in different combinations as indicated (0.8 µg each). After electrophoresis under non-denaturing conditions gel was stained with coomassie.

Formation of protein complexes of SSs and BEs or phosphorylase were reported in several species [37]–[42], [49]. Protein extracts from *be* mutants (*Atbe3*, *Atbe2*, *Atbe3/2*) were analysed for SS activity, to investigate if BEs contribute to the observed glucan acceptor independency of SSI. SSI activity was observed in *Atbe3* and *Atbe2* without addition of a glucan acceptor, but was lacking in extracts of *Atbe3/2* double knock-out plants (Fig. 3A). For all *be* knock-out lines the SSI activity was clearly detectable when glycogen was co-polymerized in the gel (Fig. 3B). These results show that the observed SSI (without addition of a glucan acceptor) activity clearly depends on the presence of either one of the two BE isoforms. Additionally, citrate stimulated this reaction, as omitting citrate resulted in weaker activity (see Fig. S2 in File S1). To analyse the effect of BEs on the SSI activity, protein extracts of *Atbe3/2* (SSI isoform is present) and *AtssI* (both BE isoforms are present) were mixed in different ratios directly prior to electrophoresis (Fig. 4A and B). This demonstrated that SSI dependent activity can be visualized if BE (present in *AtssI* mutant) was added to SSI (present in *Atbe3/2* mutant). Nevertheless, the amount of SSI protein was important for staining intensity, as more SSI enzyme in the mixture resulted in a more intensive activity band. This cooperation was independent of the gel concentration (between 7.5–9.5% AA/bisA; Fig. 4A–B). We further analysed the mobility of the proteins under different gel concentrations, as identical migration distances would be an indication for a possible protein complex formation. Therefore, we performed a two-dimensional electrophoresis composed of native Page and subsequent SDS-PAGE (Fig. 4C). The position of the proteins differed, depending on gel concentration of the first dimension (native PAGE). Proteins co-migrated and were quite close to the running front at low gel concentrations. However, the different proteins (BE2, BE3, and SSI) were clearly separated at higher gel concentration (9.5%; Fig. 4C). The relative distance of the proteins to each other was unchanged also when analysing the mutants *AtssI*, *Atbe3* and *Atbe2*, respectively (see Fig. S3 in File S1). Similarly, coomassie staining of native PAGE revealed that the mobility of rSSI, rBE2 and rBE3 were not affected by mixing the proteins (Fig. 4D). All three recombinant proteins migrated as distinct bands (Fig. 4D); although in some experiments rSSI was closely to rBE2 and rBE3 protein bands (data not shown). However, SSI dependent activity was restored when recombinant enzymes were mixed prior electrophoresis (Fig. 4E) showing that the proximities of rBE3 and rBE2 to rSSI were sufficient for a functional interaction. Furthermore, it is clearly visible that the relative position of the resulting activity was slightly different for rBE2 and rBE3, as rBE2 revealed a shift to reduced and rBE3 to an increased migration distance compared with the situation when both enzymes were available. Thus, the observed activity bands reflected the relative position of the involved BE. However, the relative positions of rSSI and rBE2 were slightly different to that of plant extracts (compare Fig. 4C and D). Alterations in the mobility of the proteins could be related to the His-tag, potential posttranslational modifications of the plant proteins or potentially endogenous bound carbohydrates.

However, for both enzyme reactions, SSI and BE, a glucan acceptor is necessary in a strict sense. Therefore, it is reasonable to predict that endogenous glucans were bound to the enzymes.

**Figure 5:**
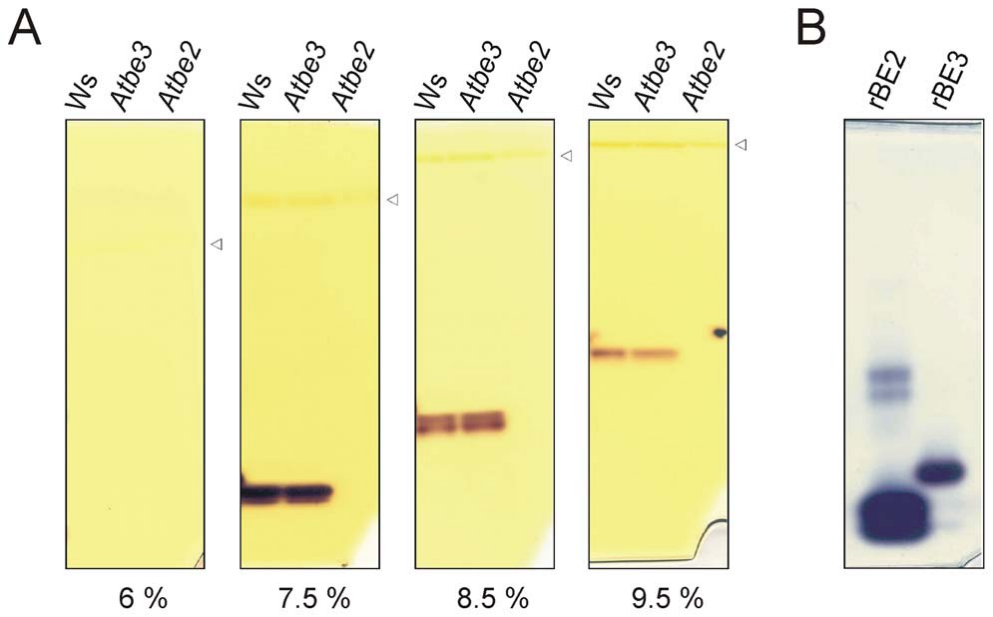
BE activities in native PAGE. 20 µg of protein extracts were electrophoretically separated under native conditions using different gel concentrations (A). Gels were incubated with phosphorylase a in presence of glucose-1 phosphate overnight at room temperature and finally stained with iodine. (B) BE activity based on stimulation of phosphorylase a was also tested for recombinant proteins (0.3 µg).

BE activity in native PAGE is based on stimulating effect of branching enzyme on the phoshorylase a activity. Phosphorylase a elongates glucans in excess of glucose 1-phosphate which are concurrently branched by BE and thereby the number of non-reducing ends increases that can be further used by phosphorylase a. In leaf extracts only BE2 activity was detectable using this assay. BE3 activity was never detected, even when the gel concentration (Fig. 5A) and/or protein amounts (data not shown) were altered. Similar observations were already reported [31]. In contrast, using recombinant enzymes both rBE2 and rBE3 formed an activity band in the gel (Fig. 5B). One can speculate that endogenous glucans bound to the branching enzymes differ. The glucans related to BE3 were not a suitable substrate for phosphorylase a, but the glucans bound to rBE3 were sufficient. Interestingly, in Arabidopsis leaf extracts BE3 was more abundant than BE2, as revealed by immuno quantification using the recombinant proteins as standards (data not shown).

### Carbohydrates observed in protein preparations of rSSI, rBE3, and rBE2

Further, we focused on the recombinant enzymes (rSSI, rBE2, rBE3) as the complexity in leaf protein extracts is much higher. The recombinant proteins were denatured and treated either with amyloglucosidase or with isoamylase for glucose measurement and for analysis of the chain length distribution (CLD), respectively. Each of the protein preparations contained glucans although the amount of glucosyl residues were very low (nmol per mg protein). rSSI preparations contained in average 3 to 4 times less glucosyl residues than rBE2 and rBE3, respectively (Table 1). No glucose was detectable when preparations were not pre-treated with amyloglucosidase (data not shown). Furthermore, when the proteins were expressed in a glycogen deficient *E. coli* strain (*ΔglgCAP*; [16]); glucosyl residues were detected in some but not in all preparations (Table 1). However, in this *E. coli* strain only the formation of glycogen is defective but not the formation of maltodextrins [50], [51]. Therefore, smaller glucans inside the bacterial cell could interact with and finally bind to the respective proteins and can be co-purified. Theoretically, each BE molecule in average contained two (e.g. maltose) to four (e.g. maltotetraose) glucosyl residues, when ratio of glucosyl residues per molecule enzyme was calculated for enzymes expressed in non-mutated BL21(DE3) *E. coli* strains (Table 1). In contrast, not every rSSI enzyme molecule contained a glucan or even glucose. The reason that rBEs preparations contained a higher glucan content than rSSI preparations can be related to the existence of N-terminal carbohydrate binding modules (CBM 48) in these enzymes (www.kegg.jp). Protein bound glucans were analysed using HPAEC-PAD. For all protein preparations glucans were detected following isoamylase treatment. For better evaluation glucan chains were grouped in short (DP 2–7), intermediate (DP8–13), and long (DP 14–20) chains (for complete CLD profiles see Fig. S4 in File S1). Note, CLD analyses were limited to DP 20 but also longer chains were detected and account for less than 2% (data not shown). In Table 1 the relative proportion of DP-groups were summarized. Obviously, glucans of both proteins rBE2 and rBE3 contained predominantly short chains that were more accented in rBE3. In contrast, rSSI contained slightly but not significantly more intermediate chain than the shorter DPs. In summary, CLD profiles revealed that the endogenous glucans bound to the enzymes were branched and large. As a consequence, only few enzyme molecules contained glucans or several enzyme molecules were associated with the same endogenous glucan.

**Table 1:** Characterization of endogenous carbohydrates extracted from recombinant enzyme preparations.

	nmol glucose mg protein^−1^		molecular ratio^a)^	relative area [%]		
	BL21(DE3)	BL21(DE3)	BL21(DE3)	DP 2–7	DP 8–13	DP 14–20
		*ΔglgCAP*				
rSSI	9±5.8	1.5; 14.5	0.6±0.4	39±9.6	47±4.9	14±5.7
rBE2	29±18.2	n.d; 2.4	2.5±1.6	51±1.8*	38±2.3**	11±0.8
rBE3	41±18.5	n.d; 9.4; 23.4	3.8±1.7	70±13.0**	23±9.3***	7±3.8

Recombinant proteins were denatured and treated with amyloglucosidase prior to glucose estimation. Heterologous expression were either performed in *E. coli* BL21(DE3) strains or in glycogen deficient *ΔglgCAP* cells with the same genetic background. For characterization of endogenous glucans enzyme preparations expressed in BL21(DE3) were denatured and treated with isoamylase. Debranched products were analysed by HPAEC-PAD. For evaluation of data different chain length peaks were grouped. Only DP populations (DP2–7, DP8–13, and DP14–20) were tested for significance among the enzyme preparations.

Differences were significant with * P = 0.05, ** P = 0.02 or *** P = 0.01 (rSSI with rBE2 and rBE3). Differences (DP-2-7 and DP8–13) between rBE2 and rBE3 were significant with P = 0.05. Average values and standard deviation of four independent experiments for each preparation are given. a) Ratio between nmol glucose and nmol protein. n.d. – not detectable.

### In *in vitro* assays rSSI formed branched glucans with both rBE2 and rBE3, respectively

**Figure 6:**
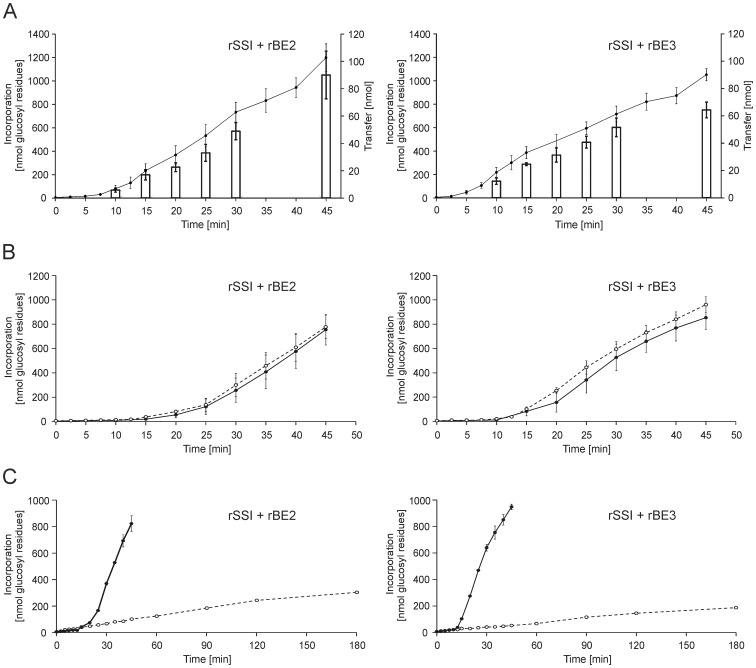
Interaction between rSSI and rBE2 and between rSSI and rBE3. rSSI/rBE2 (0.07 nmol each) and rSSI/rBE3 (0.35 nmol each) were incubated with 1,500 nmol ADPglucose in a total volume of 50 µl. (A) Reaction was performed without any external applied glucosyl acceptor. Incorporation of glucosyl residues were estimated after amyloglucosidase treatment. Additional to measured incorporation of glucosyl residues, reducing ends were estimated after isoamylase treatment (open bars and right axis). (B) Proteins of rSSI, rBE2 and rBE3 expressed in *E. coli ΔglgCAP* deletion mutant were incubated without any external glucan applied (closed circles with solid line) or incubated in presence of 25 nmol (glucose equivalents) maltodextrins with range of DP6 to DP30 (maximum peak DP15) (open circle with broken line). (C) rSSI was incubated in presence of maltodextrins (see above) either with native rBE2 or rBE3 (closed circle with solid line) or with denatured proteins (open circle with broken line). Average values and standard deviation from independent experiments with n = 6 (A), n = 4 (B) and n = 3 (C) are given.

For better understanding of the functional interaction between SSI and the two BEs we performed enzyme assays in solution containing recombinant enzymes and ADPglucose. An external glucan acceptor was not applied; except for some control experiments maltodextrins were used. Protein amounts of rSSI and rBEs were adjusted to comparable product detection values. In principle, glucosyl incorporation determined following amyloglucosidase digestion as degree for rSSI activity, and reducing ends, estimated after isoamylase treatment as degree of branching activity, were measured at different time points. The transfer reactions catalysed by the two rBEs were very similar and glucosyl incorporation increased with time (Fig. 6A). The initial glucan concentration was quite low (0.23 nmol in average for rSSI+rBE2 and 0.16 nmol in average for rSSI+rBE3), as in the reaction any external glucan acceptor was omitted. For both reactions a lag phase within the first 10 to 15 minutes was monitored. Obviously, in the beginning the critical concentration of the glucan acceptor was too low for optimal interaction of the proteins. However, there was a clear correlation of increasing glucosyl incorporation and transfer via rSSI and rBEs, respectively. Glucosyl residues were also incorporated using recombinant proteins expressed in *ΔglgCAP E. coli* cells, but the lag phase was longer with up to 20 to 25 minutes (Fig. 6B). In these reactions the amount of glucans at the beginning was much lower (0.04 nmol for rSSI + rBE2 and 0.06 nmol for rSSI + rBE3). Interestingly, the lag phase was still the same and the incorporation was only slightly increased when a mixture of maltodextrins (25 nmol of glucose equivalents and 1.7 nmol reducing ends) with DP 6 to 30 (maximum DP15) was added to the reaction mixture (Fig. 6B). Experiments in which glucans were added to the reaction mixture and the BEs were denatured prior incubation revealed the importance of the BEs for the observed incorporation rates (Fig. 6C). Even after several hours of incubation, glucosyl incorporation not exceeded 400 nmol. Note, as half molar amount of rSSI was applied in the reaction with denatured rBE3 the incorporation was lower than observed with denatured rBE2.

### Also rSSII, rSSIII, and rSSIV were able to interact with rBEs

**Figure 7:**
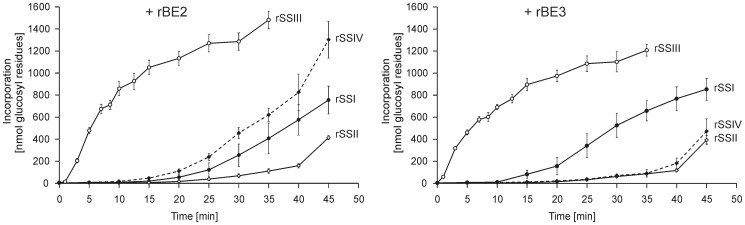
Interaction between rSSI-rSSIV with rBE2 and rBE3. 0.07-rSSIV and rBE2 each or 0.35 nmol of rSSI-rSSIV and rBE3 each were incubated with 1,500 nmol ADPglucose in a total volume of 50 µl without any external applied glucan acceptor. Incorporation of glucosyl residues after different time points was estimated after amyloglucosidase treatment. Average values and standard deviation of four independent experiments are given.

rSSII, rSSIII, and rSSIV were tested under the same conditions, to verify if the observed functional interaction is specific for SSI. Time-dependent increased incorporation of glucosyl residues was detected for both BEs (Fig. 7A and B). All different SS isoforms were able to interact with the BEs without the addition of any external glucosyl acceptor, even the efficiencies were different. Nevertheless, like shown for rSSI, it is also likely that the preparations of the other SSs contained traces of glucans, which would explain the observed activity of rSSIII in native PAGE (Fig. 2B). Comparison between the rBE2 and rBE3 reactions were difficult, as different molar amounts of enzymes were used and as a consequence the amount of endogenous glucans were altered which either influence the reaction kinetics.

### *In vitro* SSI and BEs were able to form branched carbohydrates displaying a polymodal CLD similar to that of Arabidopsis leaf starch

**Figure 8:**
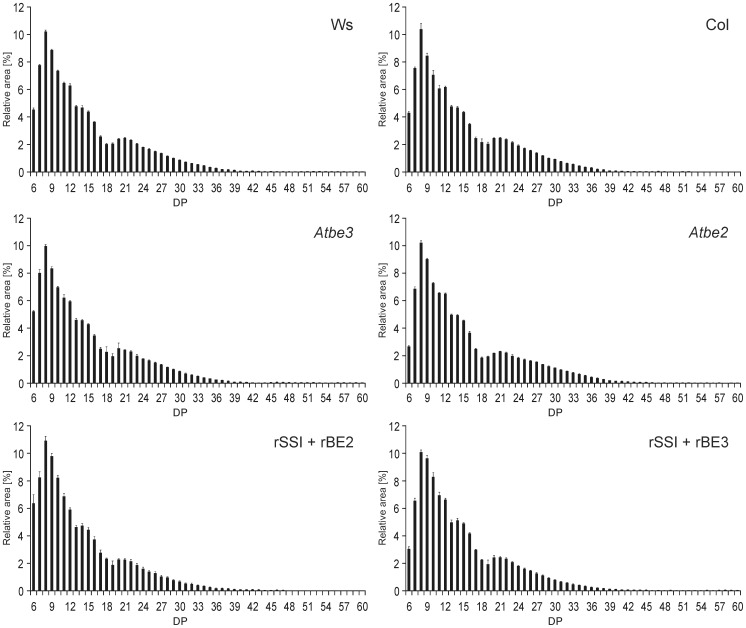
CLD profiles of glucans formed by the interaction of AtSSI with AtBE2 or AtBE3 in native PAGE. Protein extracts from Arabidopsis leaves (300 µg per cm lane) of Ws, Col, *Atbe3* and *Atbe2* were electrophoretically separated using native PAGE. Following electrophoresis gels were incubated in 1 mM ADPglucose in presence of citrate overnight at room temperature. As glucans form a milky precipitate in the gel iodine staining was omitted. Glucan chains below DP6 (DP3–5), representing only 0.8–3.5% of the total peak area, were not reproducible and therefore excluded from further analysis. Average values and standard deviation of four independent experiments are given.

SSI and the two BEs were able to interact without the presence of an external glucosyl acceptor, irrespective if the recombinant forms or proteins extracted from leaf material were used. The glucans formed in-gel were branched and stainable with iodine. Glucans were extracted from native gels and CLD profiles were recorded (Fig. 8). Glucans formed displayed similar CLDs irrespective if enzymes extracted from leaves of wild type (both Ws and Col-0) or single knock-out mutants *Atbe3* and *Atbe2* were used (Fig. 8). The DPs were polymodal distributed and can be grouped in at least three different pools. The first group between DP6 and 12 is a heterogeneous population with a main peak of DP8. Two local minima at DP 13 and DP 18/19 were prominent in all CLD profiles. Interestingly, the glucan products of rSSI/rBE2 and rSSI/rBE3 interactions showed equal CLD profiles (Fig. 8).

**Figure 9:**
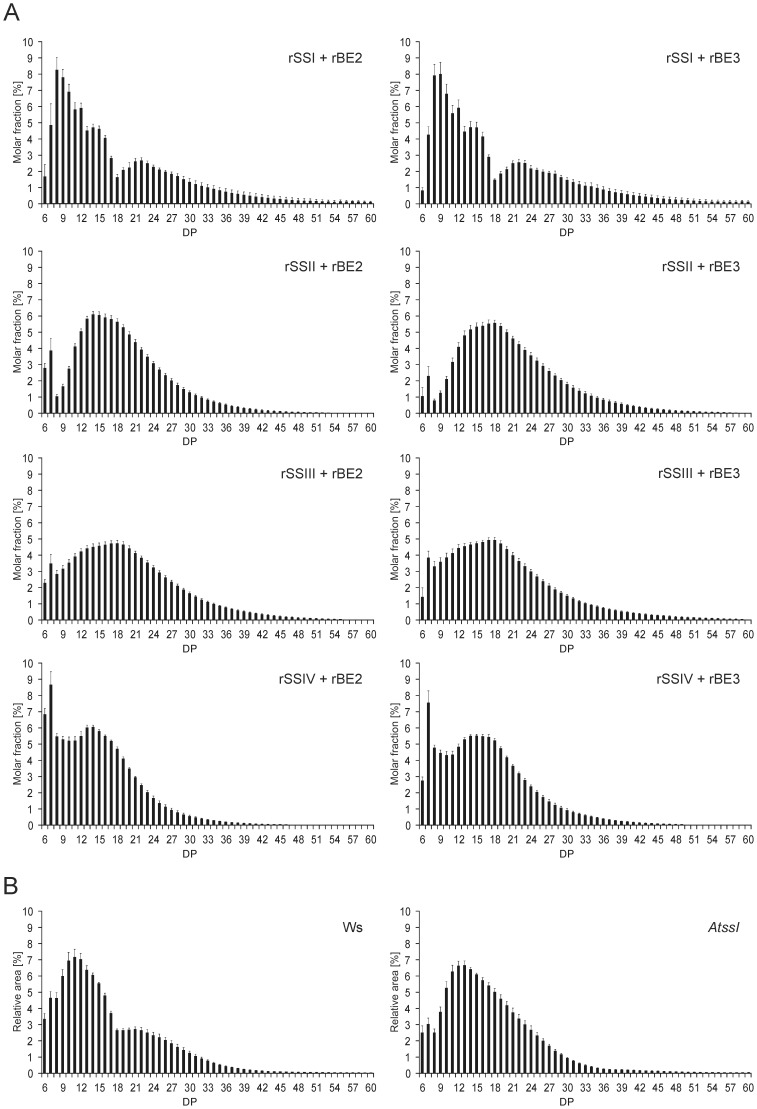
CLD profiles of both, glucans formed by the interaction of rSSI-rSSIV with rBE2 or rBE3 and of Arabidopsis starches. (A) Proteins were incubated as described for Fig. 7. At different time points (glucosyl incorporation between 300 and 900 nmol) aliquots were taken and treated with isoamylase. Glucans were labelled with APTS and separated using capillary electrophoresis. Average values and standard deviation of independent experiments with rBE2 interacting with SSs (rSSI n = 8, rSSII n = 6, rSSIII n = 5 and rSSIV n = 6) and rBE3 interacting with SSs (rSSI n = 5, rSSII n = 6, rSSIII n = 5 and rSSIV n = 6) are given. Glucan chains shorter than DP 6 were in parts irregular and below 2% of the total peak area and therefore were excluded from the data. (B) Starch granules from Arabidopsis leaves were extracted from plants harvested at the end of light period. CLD profile of debranched starch from wild type (Ws) and *AtssI* knock-out line were analysed by HPAEC-PAD. Average values and standard deviation of four independent experiments are given.

CLD profiles of glucans formed by rSSI/rBE interactions extracted from native gel (Fig. 8) and those formed in solution (Fig. 9A) displayed the same characteristic features regarding polymodal distribution including most abundant chains and prominent local minima (DP8–9, DP13 and DP18/19) but differed in the distribution of molar fractions. The latter is most likely related to the lower amounts of glucans in total together with the more difficult isolation of long chains from native gels and the lower sensitivity for long chains by HPAEC-PAD.

However, we tested whether all SS isoforms in combination with either of the two BE enzymes were able to form this characteristic CLD profile. Glucan products were analysed from different time points during the reaction representing 20 to 60% of ADPglucose consumption (depicted by standard deviation, Fig. 9A). Each SS isoform revealed a characteristic CLD profile when interacting with BEs. However, none of the interactions led to a polymodal distribution as observed for rSSI/rBE2 and rSSI/rBE3. When comparing the CLD profiles between rBE2 and rBE3 reactions, slight differences were observed for the interaction with rSSII or rSSIV indicating a shift to shorter chain lengths if rBE2 was present, whereas for rSSIII an opposite tendency to shorter chains was observed when rBE3 was present. Maximum chain length of glucans formed by rBE3 with the rSSII-IV isoforms was in average five glucosyl residues longer than those chains formed by interactions with rBE2. No such differences were observed for the interaction of rSSI with BEs. Interestingly, a similar polymodal distribution with local minima at DP 13 and DP 18 was observed for CLD of starch extracted from Arabidopsis wild type plants, whereas the polymodal distribution disappeared in starch of *AtssI* knock-out plants (Fig. 9B).

## Discussion

SSs and BEs are the key enzymes responsible for the formation of the α-1,4 and α-1,6 glycosidic bonds within the amylopectin molecule. We showed that SSI activity requires the existence of at least one BE activity (Fig. 3, 4, Table S2 in File S1). The migration distances of SSI and the BEs in native PAGE were not identically but were sufficient for the functional interaction resulting in glucan formation (Fig. 4). Thus, the interaction of SSI and BEs seems not to be a result of a protein-protein association. However, we cannot exclude the possibility that the proteins form a complex *in vivo* that was unstable under conditions of native PAGE. Nevertheless, no protein complex formations were observed using various other gel systems and e.g. blue sepharose affinity chromatography (data not shown). The stimulating effect of BE on SS activity was frequently observed [52], [53]. BE activity generates both additional non-reducing ends and glucan chains with shorter DP, favouring elongation activities of SSs. The observed interaction of Arabidopsis SSI and BEs were stimulated by citrate (Fig. 3, 4A and B), known to promote functional interactions [52], [53]. Despite this, omitting citrate did not prevent this interaction (see Fig. S2 in File S1), as only the rate of glucan formation was delayed (see Fig. S5 in File S1).

In purified SS or BE preparations from spinach and maize endogenous glucans were identified and it was concluded that those glucans served as a glucosyl donor under reaction conditions, lacking external added glucans [52], [53]. Glucans (ranging from 9 to 41 nmol glucose mg^-1^ protein) in rSSI, rBE2 and rBE3 from Arabidopsis were also detected (Table 1). Even the expression in a glycogen deficient *E. coli* strain did not prevent the association of glucans to the proteins (Table 1). Furthermore, we showed that the glucans bound to the heterologously expressed proteins were branched and revealed characteristic CLD profiles with chain lengths from DP 2 to 20 (or even longer; Table 1,Fig. S4 in File S1). The glucans in case of plant derived proteins are probably different compared to the recombinant proteins, as BE3 activity was not detected in a phosphorylase a based assay, whereas rBE3 showed activity (Fig. 5A and B). However, in plant extracts from *Atbe2* mutant BE3 was able to interact with SSI (Fig. 3, 4A and B). The glucan content in rSSI was in average lower compared to rBE2 or rBE3. As no activity of SSI was detected without external applied glucans or addition of BEs (containing glucans), the glucans bound to rSSI and to SSI from the *be* double mutant were inefficient for elongation, either due to the low amount or internal structure of the glucans. Therefore, it is reasonable to assume that the glucans bound to the BEs served as initial glucosyl acceptor for the interaction.

The CLD profiles of glucans formed by the various SS/BE combinations in-gel (Fig. 8) as well as in enzyme assays in solution (Fig. 9) point to at least two important conclusions. First, the BE isoform added to the SSs is of minor relevance, as the resulting glucan structures were similar. This also includes that the structure of the initial glucosyl acceptor (bound glucan) did not dominate the final glucan structure. Interestingly, knock-out of a single branching enzyme in Arabidopsis resulted in only weak effects on amylopectin CLD [31]. Thus, enzymatic redundancy could explain the observed similarities in glucan CLD profiles between rSS/rBE2 and rSS/rBE3 interactions.

Second, the structures of the formed glucans were dependent on the SS isoform applied. Furthermore, also the rates of glucan formation differ dependent on SS isoform applied (Fig. 7). In general, the interaction of BE and SS is limited by the SS activity, as glucan formation is driven by the incorporation of glucosyl residues from ADPglucose as glucosyl donor. Any limiting SS activity would lower incorporation rates even when BE is in excess. Conversely, the ability of BE to generate additional acceptor chains favours SS action presupposed that generated lengths of chains are suitable for elongating action.

The surplus or the reduction of rBE molar amount by 25% did not led to any significant differences in incorporation rates for both SSI/BE2 and SSI/BE3 interaction (Fig. S6 in File S1). Thus, the BE activities were not limiting factors for the glucan formation. Interestingly, only rSSIII activity was visible in native PAGE when any external glucan acceptor was omitted (Fig. 2B). This activity was probably related to an appropriate amount and structural efficient endogenous glucan bound to rSSIII and can therefore partly explain the effective interaction with rBEs (Fig. 7). Conclusively, different incorporation rates observed for SS isoforms interacting with rBE2 or rBE3 were not only related to different kinetic properties of the enzymes but also to expression/purification system together with different amounts of endogenous bound carbohydrates.

CLD pattern of glucans formed by rSSI/rBEs interactions were quite different to all others, as they displayed a polymodal distribution of chains (Fig. 8 and Fig. 9A). Interestingly, the glucans formed by the interaction of SSI with the BEs displayed the complete set of chains (A, B1, B2, B3 chains) that are required for establishment of cluster and inter-cluster structures according to Hizukuris model [8], [9]. It was supposed that SSI is unable to generate glucan chains long enough for branching, as SSI did not elongate glucan chains longer than DP20 [14]. This was concluded from biochemical studies with recombinant SSI [26] and purified BE [54], [55] from maize. Furthermore, BEs cannot act on donor-chains shorter than DP12 [54], [55]. Therefore, it was assumed that SSI and BE are unable to interact in starch synthesizing processes. However, we showed that SSI and BE interact. Glucan chains smaller than DP20 generated by SSI must be sufficient for BE action, if SSI is unable to elongate chains longer DP20. Thus, depending on branch positions available chain lengths for SSI are short, assuming that branching points have terminating effects on chain length recognition by SSI. However, only for the interaction of SSI and BEs polymodality was observed that must be related to protein characteristics of the SSI isoform.

A polymodal distribution of amylopectin glucan chains is characteristic for Arabidopsis starch [14]. The most abundant chains of the *in vitro* SSI/BE interaction displayed a DP of 8 and 9 instead of DP12 found in amylopectin (Fig. 9A and B). Similar to wild type starch the glucans revealed a peak around DP 21 together with two local minima at DP13 and DP18 but these were more pronounced (compare CLDs in Fig. 9A and B). We conclude that the interaction of SSI/BE is responsible for establishing a specific population of chains that are typical for *Arabidopsis thaliana* amylopectin, including chains longer than DP 40 (see results, Fig. 9A and B). Consistently, several single, double, and triple mutants of SS isoforms displayed specific alterations in the CLD profiles of amylopectin compared to wild type [14]–[19], but only the lack of SSI resulted in disappearance of the polymodal distribution (Fig. 9B). Furthermore, peak modality of amylopectin glucan chains of *ssII/ssIII* double mutant [19] was most similar to the glucans synthesized by SSI/BEs *in vitro*. Consequently, the difference plots obtained by subtraction of the CLD profile of *AtssI* mutant starch from those of the SSI/BEs glucans and wild type starch showed comparable alterations of glucan chains (Fig. S7 in File S1).

According to our results SSI/BE interaction is responsible for establishment of the local minima at DP18 and DP13. This SSI/BE specific glucan pattern was also found in storage starches from different biological origins, like barley [23], [56]–[58], cassava [59], [60], and species from the *Triticum-Aegilops* group [57], [61] including wheat [22], [57], [62]–[67]. In starches of maize [24], [68], [69], potato [70] or rice [71] the SSI/BE specific glucan pattern was usually not detectable but becomes evident when one or more other SS isoform(s) (SSII, SSIII) was/were lacking. Interestingly, deficiency of SSIII activity was accompanied by an increase of SSI activity in barley, maize, potato, and rice [70], [72]–[74]. For rice it was discussed that elevated SSI activity is responsible for establishment of altered proportions of glucan chains between DP6 and 25 [71], [72].

Additionally, modelling of CLD data revealed that two local minima at DP13 and DP18 with slope of DP13–17 and DP18–22 correlates with functionality of SSIIa in rice and wheat [75].

Our work provides evidences that the typical modality including the two local minima at DP13 and DP18 observed in starches of several plant species are directly linked to interaction of SSI isoform with BE. Synergistically and/or concurrently interaction of BEs with other SS isoforms results in decreased perception of the typical SSI related CLD profile.

## Supporting Information

File S1 **Supporting information including tables S1–S2 and figures S1–S7 can be found in File S1. Table S1.** Cloning of *AtSSI-AtSSIV*, *AtBE2*, and *AtBE3*. **Table S2.** rSSI activity in radiolabelled assays. **Figure S1.** SSI activity throughout the diurnal cycle. **Figure S2.** SS activities of *be* knock-out plants. **Figure S3.** Mobility of SSI, BE2, and BE3 protein in native PAGE using different knock-out lines. **Figure S4.** CLD profiles of glucans extracted from rSSI, rBE2, and rBE3 preparations. **Figure S5.** Comparison of rSSI/rBE3 interaction in presence and absence of citrate. **Figure S6.** Effect of BE amount on rSSI/rBEs interaction. **Figure S7.** Difference plots of CLDs from glucans generated by SSI/BE interaction in-gel and Arabidopsis starch.
(PDF)
