# Supplementary material for: Analysis of the Functional Interaction of Arabidopsis Starch Synthase and Branching Enzyme Isoforms Reveals that the Cooperative Action of SSI and BEs Results in Glucans with Polymodal Chain Length Distribution Similar to Amylopectin
Source: PLoS One. 2014 Jul 11;9(7):e102364. doi: 10.1371/journal.pone.0102364 (PMC4094495; doi:10.1371/journal.pone.0102364)
Supplement: File S1 — Supporting information including tables S1–S2 and figures S1–S7 can be found in File S1. Table S1. Cloning of AtSSI-AtSSIV, AtBE2, and AtBE3. Table S2. rSSI activity in radiolabelled assays. Figure S1. SSI activity throughout the diurnal cycle. Figure S2. SS activities of be knock-out plants. Figure S3. Mobility of SSI, BE2, and BE3 protein in native PAGE using different knock-out lines. Figure S4. CLD profiles of glucans extracted from rSSI, rBE2, and rBE3 preparations. Figure S5. Comparison of rSSI/rBE3 interaction in presence and absence of citrate. Figure S6. Effect of BE amount on rSSI/rBEs interaction. Figure S7. Difference plots of CLDs from glucans generated by SSI/BE interaction in-gel and Arabidopsis starch. (PDF) [file pone.0102364.s001.pdf]

Analyses of the Functional Interaction of *Arabidopsis* Starch Synthase and Branching Enzyme Isoforms Reveals that the Cooperative Action of SSI and BEs Results in Glucans with Polymodal Chain Length Distribution Similar to Amylopectin

Henrike Brust, Tanja Lehmann, Christophe D'Hulst, Joerg Fettke

Supporting Information

Tables S1-2 and Figures S1-S7

Tables S1-2

Table S1. Cloning of *AtSSI-AtSSIV*, *AtBE2*, and *AtBE3*.

	transit peptide	first-strand primer	forward primer	reverse primer
AtSSI (At5g24300)	49	5'-GCTGACATAGGGAGGGTCCATGAAAACCCA -3'	*Eco*RI-linked primer<br>5'-GAATTCGTCTTCTTCCTTCTCCGGTGACTC -3'	*Xho*I-linked primer<br>5'-CTCGAGGCTGACATAGGGAGGGTCCATGAA -3'
AtSSII (At3g01180)	55	5'-CCAATGATACTTAGCAGCAACAAGAACTTC -3'	*Eco*RI-linked primer<br>5'-GAATTCGTGTGTGAGCCGCGTCGAGGCTTC -3'	*Xho*I-linked primer<br>5'-CTCGAGCCAATGATACTTAGCAGCAACAAG -3'
AtSSIII (At1g11720)	37	5'-CTTGCGTGCAGAGTGATAGAGCTCAAGATA -3'	*Eco*RI-linked primer<br>5'-GAATTCGGGAAGTGCTCAGAAAAGAACTCA-3'	*Xho*I-linked primer<br>5'-CTCGAGCTTGCGTGCAGAGTGATAGAGCTC -3'
AtSSIV (At4g18240)	42	5'-CGTGCGATTAGGAACAGCTCTTGCTCTGGA -3'	*BamH*I-linked primer<br>5'-GGATCCGTGTAAGATGCGACAACAACGTGG -3'	*Xho*I-linked primer<br>5'-CTCGAGCGTGCGATTAGGAACAGCTCTTGC -3'
AtBE2 (At5g03650)	61	5'-ATCGTGGTTTGCTAAAGCATAAACCACGGC -3'	*BamH*I-linked primer<br>5'-GGATCCGGCTTCTGAGAAAGTCTTAGTACC -3'	*Xho*I-linked primer<br>5'-CTCGAGATCGTGGTTTGCTAAAGCATAAAC -3'
AtBE3 (At2g36390)	37	5'-AACATCTTCGGGTAACAGGCCTATGGGGAC -3'	*BamH*I-linked primer<br>5'-GGATCCGTTCTCTCTGAGGAAGGACTCTCG -3'	*Xho*I-linked primer<br>5'-CTCGAGAACATCTTCGGGTAACAGGCCTAT -3'

Table S2. rSSI activity in radiolabelled assays.

2 µg of rSSI was incubated in a total volume of 50 µl 2 mM ADPglucose (0.02 µCi ADP-[U-^{14}C]glucose) and 0.025 % (w/v) bovine serum albumine. Several buffers were tested (each 10 mM and pH 8.0): Tricin/NaOH, Tricin/NaOH with 5 mM K-acetate and 0.4 mM EDTA, Tris/HCl, HEPES/NaOH, ammoniumbicarbonate and Glycine/NaOH (pH 8.4, 2 mM EDTA).
All experiments were performed with (6 mM maltose) or without an external glucan acceptor.
Activity was tested in the presence or absence of 500 mM Na-citrate pH 8.0. At several time points aliquots were taken and denatured at 95 °C for 4 minutes. Samples were analysed by thin-layer chromatography following phosphor imaging (Hejazi *et al.* 2008).
n.d. - not detected

maltose	–		+	
citrate	-	+	-	+
0.5 h	n.d.	n.d.	11 ± 2	17 ± 3
1 h	-	-	60 ± 2	91 ± 2
2 h	n.d.	n.d.	94 ± 3	95 ± 7

Figures S1-7

Figure S1

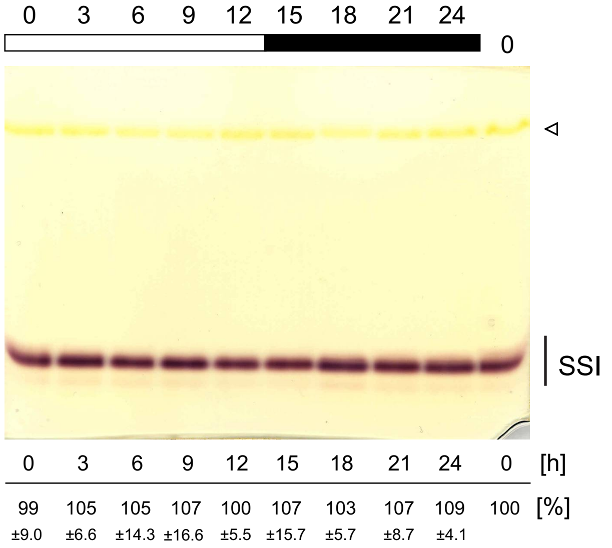

Figure S1. SSI activity throughout the diurnal cycle.

Proteins (60 µg) extracted from *Arabidopsis* wild type leaves were electrophoretically separated under non-denaturing conditions and gels were incubated with 1 mM ADPglucose in presence of citrate. Intensity of activity bands of iodine stained gels were quantified using AIDA software. Plant extract of time point 0 (beginning of light period) was loaded twice and intensity of the band in the right part of the gel was set to 100 %. Values for relative intensity in % of the samples are given. Standard deviations of four biological replicates are given.

Figure S2

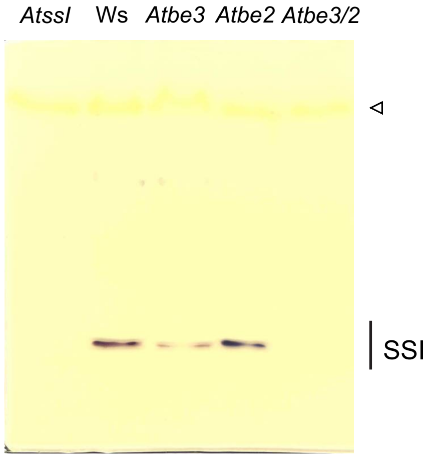

Figure S2. SS activities of *be* knock-out plants.

Extracted proteins (85 µg each) from *Atbe3*, *Atbe2*, *Atbe3/2,* wild type (Ws), and *Atssl* knock-out line were electrophoretically separated under non-denaturing conditions. Glucan free gel was incubated with 2 mM ADPglucose but citrate was omitted. After incubation overnight at room temperature gels were washed with water and stained with iodine solution. Open triangle indicates the position of rubisco.

Figure S3

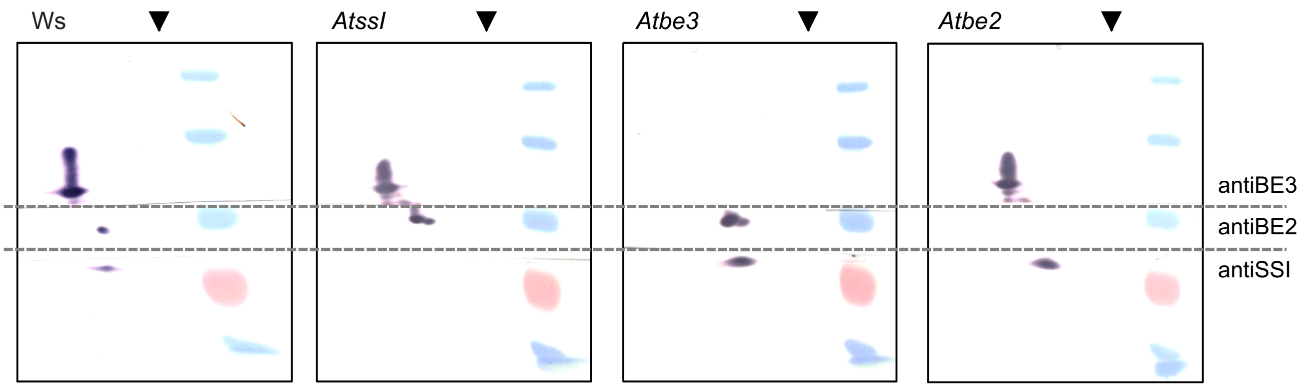

Figure S3. Mobility of SSI, BE2, and BE3 protein in native PAGE using different knock-out lines. Positions of proteins in native gel from wild type (Ws), *AtssI*, *Atbe3* and *Atbe2* single knock-out lines were analysed via immunodetection after re-electrophoresis. Native PAGE (first dimension) with 8.0 % [T] acrylamide-bisacrylamide were cut and denatured for SDS-PAGE (second dimension). Following SDS-PAGE proteins were blotted on nitrocellulose and membrane was cut in three pieces according to molecular weights of SSI, BE2, and BE3. Each of the nitrocellulose pieces were incubated with the respective antibodies as indicated. Prestained molecular mass marker as in Fig. 4C. Black triangles indicate the dye front of the gel stripes of the first dimension.

Figure S4

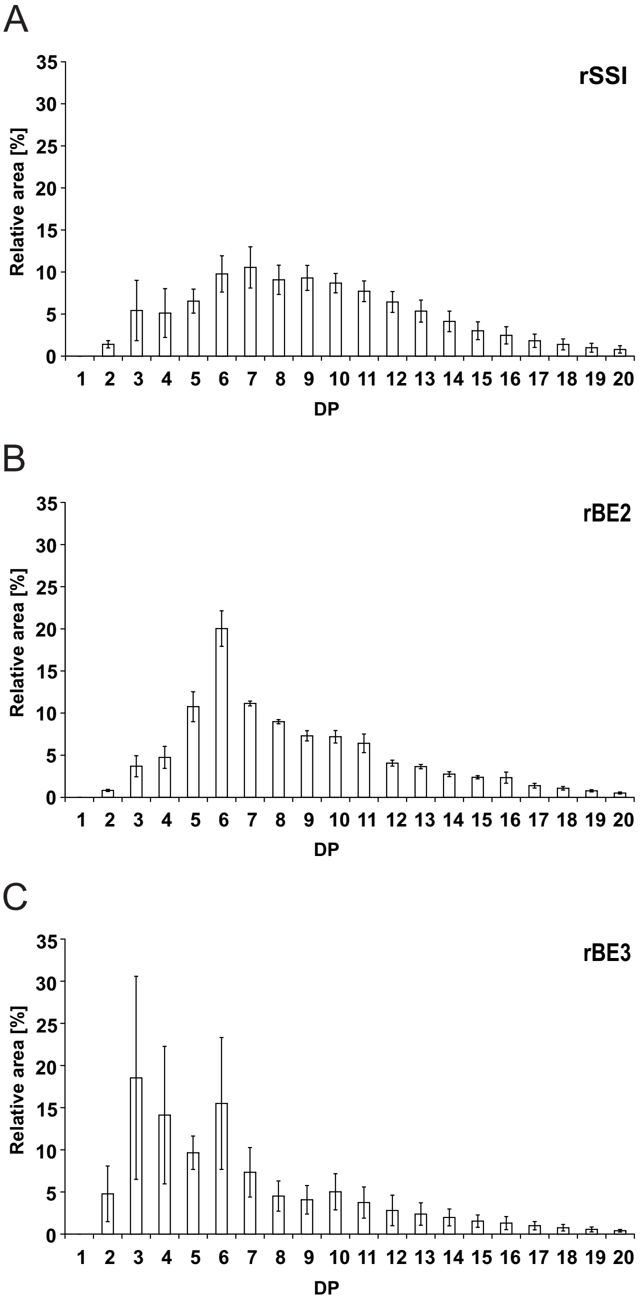

Figure S4. CLD profiles of glucans extracted from rSSI, rBE2, and rBE3.

Proteins were denatured and treated with isoamylase. The digests were analysed by HPAEC-PAD. Chains of DP2 (minimum chain length) to DP20 were used for calculation.

Figure S5

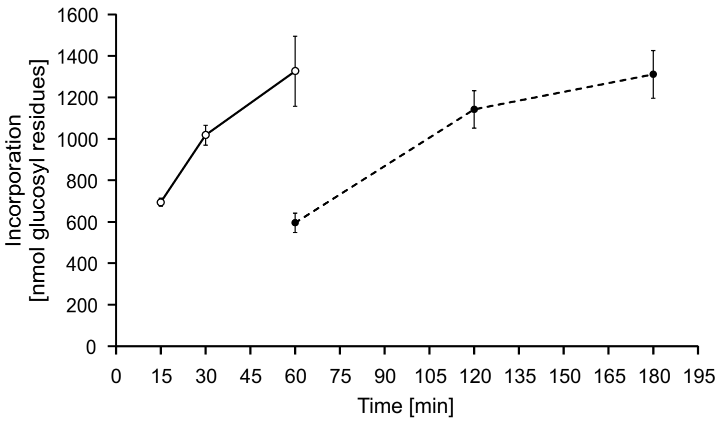

Figure S5. Comparison of rSSI/rBE3 interaction in presence and absence of citrate.
0.35 nmol of rSSI and rBE3 each were incubated with 1.5 µmol ADPglucose in a total volume of 50 µl in a buffer consisting of 25 mM Tris/HCl pH 8.0. Experiments were performed in presence of 0.5 M Na-citrate pH 8.0 (open circle, solid line) or citrate was omitted (closed circle, broken line). Incorporation of glucosyl residues were monitored by amyloglucosidase treatment and glucose determination. Average values and standard deviation of three independent experiments are given.

Figure S6

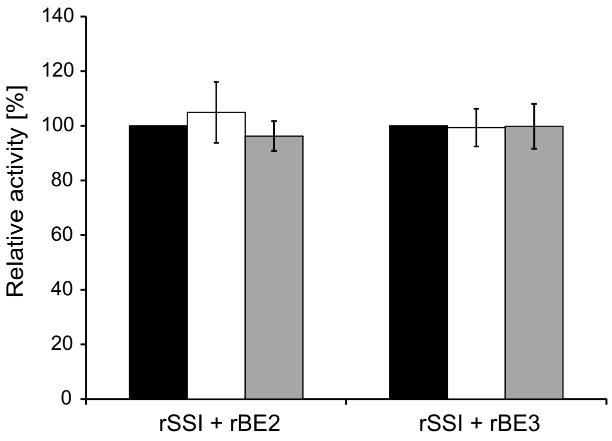

Figure S6. Effect of BE amount on rSSI/rBEs interaction.
Enzymes were incubated with 1.5 µmol ADPglucose in presence of 0.5 M Na-citrate pH 8.0 in a total volume of 50 µl. The rSSI amount was kept constant (0.7 nmol for interaction with rBE2 and 0.35 nmol for interaction with rBE3). The amount of rBEs was reduced or increased to 75 % or 125% according to the molar amount of rSSI, respectively. Aliquots at different time points (5, 10, 15, 20, 25 min) were taken and glucosyl incorporation was monitored by amyloglucosidase treatment and glucose determination. Black bars represent equimolar protein combinations (set to 100 %). Results of rSSI interacting with 75 % (white bars) or 125% (grey bars) of rBE were given. Data from the different time points are represented in the standard deviations.

Figure S7

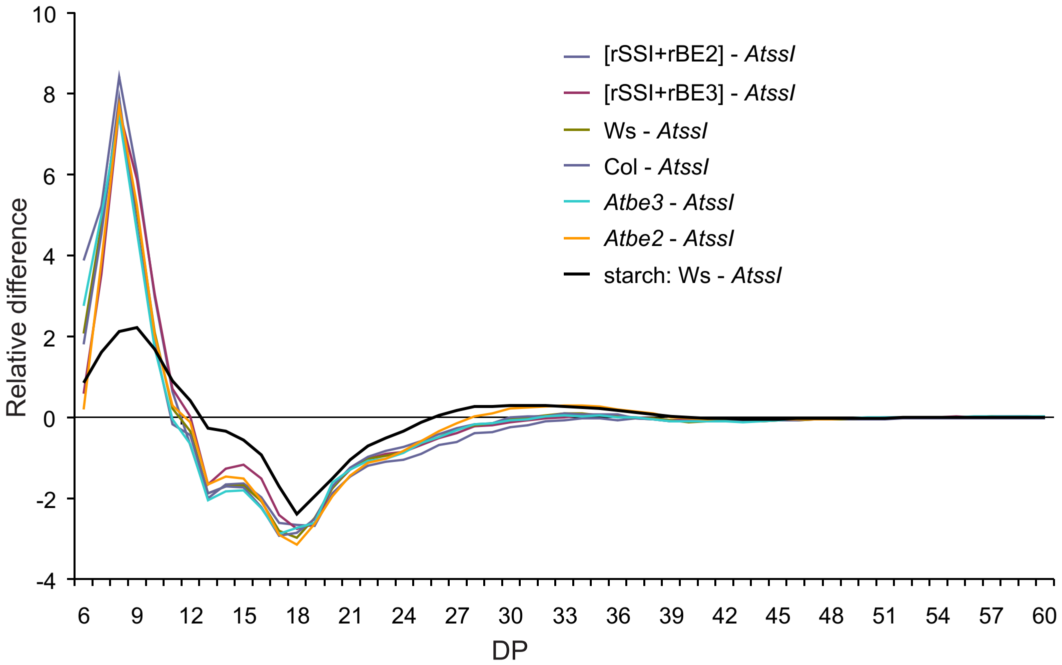

Figure S7. Difference plots of CLDs from glucans generated by SSI/BE interaction in-gel and *Arabidopsis* starch.

The CLD profile of *AtssI* mutant starch (Fig. 9B) was subtracted from CLD profiles of glucans generated in-gel (Fig. 8) and from that of wild type starch (Fig. 9B). Mean values of the respective chains were used for calculation.
